# Human resources for health and burden of disease: an econometric approach

**DOI:** 10.1186/1478-4491-9-4

**Published:** 2011-01-26

**Authors:** Carla Castillo-Laborde

**Affiliations:** 1 Department of Health Economics, Ministry of Health, Santiago, Chile

## Abstract

**Background:**

The effect of health workers on health has been proven to be important for various health outcomes (e.g. mortality, coverage of immunisation or skilled birth attendants). The study aim of this paper is to assess the relationship between health workers and disability-adjusted life years (DALYs), which represents a much broader concept of health outcome, including not only mortality but also morbidity.

**Methods:**

Cross-country multiple regression analyses were undertaken, with DALYs and DALYs disaggregated according to the three different groups of diseases as the dependent variable. Aggregate health workers and disaggregate physicians, nurses, and midwives were included as independent variables, as well as a variable accounting for the skill mix of professionals. The analysis also considers controlling for the effects of income, income distribution, percentage of rural population with access to improved water source, and health expenditure.

**Results:**

This study presents evidence of a statistically negative relationship between the density of health workers (especially physicians) and the DALYs. An increase of one unit in the density of health workers per 1000 will decrease, on average, the total burden of disease between 1% and 3%. However, in line with previous findings in the literature, the density of nurses and midwives could not be said to be statistically associated to DALYs.

**Conclusions:**

If countries increase their health worker density, they will be able to reduce significantly their burden of disease, especially the burden associated to communicable diseases. This study represents supporting evidence of the importance of health workers for health.

## Background

The labour force is an essential input in any productive system, and health care is not the exception. As Gupta and Dal Poz [[1], p.2] state, the 'functioning and growth of the health systems depend on the time, effort and skill mix provided by the workforce in the execution of its tasks'.

The World Health Report 2006 defines health workers as 'all people engaged in actions whose primary intent is to enhance health' [[2], p.1]. In this context, the health workforce includes health services providers (e.g. physicians, nurses, midwives, and laboratory technicians) as well as health management and support workers (e.g. accountants in a hospital, administrative professionals, and drivers).

In recent decades, worldwide concern about the shortage of health workers has been growing [3,4]. The estimated shortage is about 4.3 million doctors, nurses, midwives, and support workers worldwide [2] and is considered as a 'global health crisis' [[5], p.1984] because it affects not only developing countries but also developed countries; forcing them to implement new policies in order to train, sustain and retain the workforce.

Considering that the provision of quality health care depends on the adequate number, distribution and training of Human Resources for Health (HRH), the aforementioned shortage must be an important part not only of the health policy agenda, but also of the health research agenda, particularly taking into account the implications that it has on equity.

As Speybroeck mentioned [6], the distribution of the health workers throughout different countries is an important factor to consider when equity concerns are taken into consideration, and even though the shortage is present in nearly all countries, it affects more severely the poorest countries in the world. For instance, sub-Saharan Africa has only 4% of the health workers but 25% of the global burden of disease, while the Americas have 37% of the health workers and only 10% of the burden of disease [2].

Although the poorest countries are the most affected by the scarcity of health workers, most of the countries in the world are affected by problems related to their health workforce. The availability of an appropriate number of health workers is an important (if not the most important) issue to solve, but not the only one. The productivity of the existent resources, the appropriate skill mix (i.e. allocation throughout different occupations), the geographical distribution of the health workers according to the population needs, and the quality of the services delivered by them are just a few examples of other issues to consider, generally neglected by the decision makers. As Dussault and Dubois stated [[7], p.14], '[t]he lack of explicit policies for HRH development has produced, in most countries, imbalances that threaten the capacity of health care systems to attain their objectives'.

Migration is one of the most readily-recognised contributors to the increasing shortage in some of the world's most disadvantaged countries (i.e. 'source countries'). At the same time, it represents a way to deal with the shortage in the destination countries. Differences in salaries as well as working conditions are major incentives to migrate; therefore, a key component of health policies on human resources must incorporate financial and non-financial strategies to retain the health workers, especially in poor countries.

Gupta and Dal Poz [1], in a cross-country comparison including six countries, highlight the 'dual employment' (i.e. when the employee holds more than one position in different locations) as a factor which may represent a signal of unsatisfactory salaries. Dräger et al. [8] present a cross-country comparison of health workers' wages (i.e. physicians and professional nurses) for 42 countries, where data are available from the OWW database (i.e. International Labour Organization October Inquiry and Occupational Wages around the World), showing huge differences in average yearly wages earned by physicians and nurses between developed countries (USA being the highest) and the same professionals in poor countries. As the wage differentials have been proven to be so large between destination and source countries, Vujicic et al. [9] suggest that non-financial incentives may be more effective in order to retain health workers in their countries.

Another problem regarding human resources for health is the skill mix imbalance, which can be appreciated by the great differences in the composition of health teams throughout different countries (e.g. ratio nurses to physicians, specialists to physicians or health care management to physicians). As official data on number of specialists are not always available, a common indicator of skill mix that can be compared throughout countries is the ratio of nurses to physicians. The World Health Report 2006 [2] states that this varies between 5:1 in the World Health Organization's (WHO) African Region and 1.5:1 in the WHO Western Pacific Region.

The substitution of health workers (e.g. high-level cadres substituted by mid-level cadres) has been suggested in the literature as one of the alternatives to deal with the shortage of health professionals in poor countries at a lower cost [10-12]. However, the evidence regarding skill mix in the health care workforce, and in particular the degree of substitutability between different cadres, is still limited and mostly descriptive [13].

In any case, the availability of data on health workers and wages is one of the major current obstacles to conducting health workforce research and, therefore, also to developing appropriate health worker policies. Nonetheless, WHO is developing some projects in order to improve the availability of these data at a worldwide level (e.g. WHO Human Resources for Health Minimum Data Set, [14]).

Although it may seem clear that health workers play a fundamental role in the delivery of health interventions, and that, through this, their availability and actions have direct effect on people's health, a question that may arises from this evidence is exactly how much of the burden of disease can be explained by the density of health workers.

The purpose of this study is to conduct a cross country study in order to analyse descriptively and econometrically the relationship between human resources for health (i.e. density of health workers) and population health outcomes, focusing especially on the burden of disease (i.e. disability-adjusted life years (DALYs)), and compare these results with the results for other outcome indicators previously analysed in the literature (i.e. vaccination coverage and mortality). Finally, the analysis will be extended considering separately the DALYs of the three different groups of the burden of disease as the dependent variable (i.e. communicable, non-communicable diseases, and injuries), in order to study the possible different effects of the variable of interest (i.e. health workers) on these different groups of diseases.

The essay is organized into five sections. The second section reviews the literature, presenting some theoretical and empirical considerations regarding the relationship between health workers and population health. The third section describes the data and the methodology of the study. The fourth section presents the results and discusses the policy implications of the main findings. The final section summarises the conclusions.

### Literature review: what the literature says about the relationship between health workers and health outcomes

The World Health Statistics 2009 [15] indicate that the global average number of physicians per 10 000 people is 13. However, there is a wide range of variation between the different regions. For instance, while in the European Region the number of physicians per 10 000 populations is 32, it is just 2 in the African Region. In the case of nurses and midwives, the global average per 10 000 is 28, but again there are significant variations, ranging between 11 and 79 per 10 000 in the WHO African and European Regions respectively.

Considering physicians, nurses, and midwives, Speybroeck, et al. [16] estimate that countries with less than 2.28 health workers per 1000 people (i.e. 23 per 10 000 populations) will present problems to achieve 80% skilled coverage of births, one of the interventions considered by the Millennium Development Goals (MDG). Looking at this threshold and the average densities mentioned above, the African Region appears to be in a disadvantaged position in terms of the achievement of the MDGs [10]. In fact, it has been estimated that there is a shortage of more than 800 000 physicians, nurses, and midwives in this region [17,18].

The growing concern about health workers has represented a great incentive to develop literature in this area, especially in the context of health policies, to deal with the problems associated with the shortage or the imbalance of the health workforce. Moreover, there seems to be a consensus in the literature concerning the critical role of the human resources for health in terms of the management and delivery of health services, especially considering that they account for an important part of the health budgets in most of countries [19].

In this context of concern about the health workforce it is important to keep in mind that the main goal of any health system is to enhance population health. It cannot be denied that health workers are a key input in the productive process of health care (i.e. playing a fundamental role in the delivery of health interventions), and therefore they have a direct effect on the population health (i.e. the final outcome). However, a question that arises is how much of this 'health' can be 'explained' by the density of health workers. In order to answer this question a crucial issue is to find a measurable indicator of 'health'. Smith et al. [[20], p.4] describe the population health measures as 'measures of aggregate data on the health of the population'; for instance, life expectancy, years of life lost, avoidable mortality, or disability-adjusted life-years (i.e. DALYs).

Previous cross-sectional studies have attempted to assess the relationship between the human resources for health (e.g. density of doctors, density of health workers, and density of nurses and midwives) and the health outcomes (e.g. maternal, infant and under-five mortality rate, vaccine coverage, and coverage of skilled birth attendants).

Not only do the health outcomes considered as a dependent variable different from study to study, but so are the independent variables included (e.g. controlling for poverty, GDP, and adult literacy), in addition to the different functional forms for their econometrics analysis (for instance, logit-log [21], log-linear [22], linear regressions with arcsin and log transformation of the dependent and independent variables [23,24], logit-log and arcsine-log model [16]). Furthermore, the results from the studies come to different conclusions.

Kim and Moody [25], and Hertz and Landon [26] found no significant association between density of doctors and infant mortality; while Cochrane et al. [27] recorded an adverse association (i.e. positive) between the density of doctors, and infant and perinatal mortality.

On the other hand, more recent studies have found a positive and a significant association between the density of health workers and the health outcomes. Robinson and Wharrad [23] state a negative relationship between the density of doctors and the two dependent variables, 'infant mortality rate' and 'under-five mortality rate'. In 2001, the same authors found a negative relationship between the density of doctors and maternal mortality [24]. However, both studies also show the 'disappearing' (i.e. no statistical significance) of nurses.

Anand and Bärninghausen [22], controlling for gross national income per capita, income poverty and female adult literacy, present a negative association between the density of doctors and maternal, infant, and under-five mortality. The coefficient for the density of nurses was negative and significant just in the case of maternal mortality, with no significance in other cases.

Anand and Bärninghausen [21], controlling for gross national income per capita, female adult literacy, and land area, present a positive relationship between the density of aggregate health worker (i.e. including doctors and nurses) and the coverage of three kinds of vaccination (i.e. MCV, DTP3 and polio3). When including health workers separately, the density of nurses was significantly associated with the three dependent variables, but the effect of physicians on the dependent variables was found to be not significant.

Finally, Speybroeck, et al. [16], controlling for income poverty, GDP and female literacy, found a positive relationship between the density of aggregate health workers and the coverage of measles immunization and skilled birth attendants. In the case of disaggregate densities, they found a significant association between the density of physicians and the dependent variables, while the relationship was found not to be significant in the case of nurses.

All the studies mentioned above have considered the health outcomes related to mortality, the coverage of a particular disease immunization or the coverage of skilled birth attendants. Although all of these health outcomes are related to the Millennium Development Goals, in recent decades interest has grown in more comprehensive indicators of population health, capable of combining mortality and morbidity [28]. In this context, a measure of the overall burden of disease such as DALYs (i.e. the aggregation between YLL (years of life lost), and YLD (years lived with disability)), which can capture the impact of fatal as well as non-fatal diseases, is interesting to investigate as a health outcome or as a dependent variable.

As it has been stated by the literature, these kinds of health indicators (e.g. DALYs) may be influenced by factors outside the health care system [28], an idea captured by the concept of social determinants of health, or social determinants of health inequalities [29,30]. This implies that an analysis on the effect of any input (e.g. health workers) or the characteristics of the health care system on an indicator such as DALYs must control for other factors such as socioeconomic variables.

## Data and methods

The data from different public sources were collected in order to conduct a cross country study to analyse descriptively and econometrically the relationship between the human resources for health and the health outcomes. Previous studies have analysed this relationship considering the health outcomes such as child mortality or vaccination coverage. However, this study is focused particularly on the burden of disease (i.e. DALYs) as the health outcome of interest.

The availability of data on DALYs, as well as for health workers (i.e. physicians, nurses, and midwives), for all the WHO Member States allowed not only the analysis of the statistical relationship between these two variables, but also the inclusion of other variables, for instance the mix between professionals (i.e. ratio doctors/nurses and midwives) which is also considered in the literature as an important determinant of the health outcomes. The analysis also considers health expenditure as a percentage of gross domestic product (GDP) and socioeconomic variables in order to control and capture the effect of other factors that may affect health.

The data on the number (and density per 1000 populations) of physicians, nurses, and midwives were obtained from the World Health Statistics 2009 [15]. These data are part of the global WHO health workforce database and are derived from multiple sources such as administrative records, establishment census/surveys, labour force or other household surveys, national population, and housing censuses. Dal Poz et al. [31] present detailed information on the sources, limitations, and distribution of these data.

The data on the nurses and midwives are presented in an aggregated way in the report. As Anand and Bärnighausen mentioned [22], in some countries these two categories exist separately but have similar training and overlapped tasks, while in other countries midwives do not exist as a separate category, therefore it may be better to include them in an aggregated manner. The data on the number of other cadres (i.e. dentistry personnel, community health workers, and other health service providers) are presented in the report. However, as data were missed for several countries, and also considering that previous studies focused just on the three categories mentioned above, the other cadres were not included in the analysis.

The total expenditure on health as a percentage of GDP (2002) was extracted from the Global Health Atlas [32]. Following Xu et al. [33], this variable was included as a proxy of the relative degree of health system capacity.

The socioeconomic variables included in the analysis are the GDP per capita, the percentage of rural population with access to clean water, the GINI coefficient, and the income share held by the lowest 10% of the population. The former was included as a measure of income, the second as a proxy of absolute poverty, and the remaining variables as a measure of income distribution. The data for the year 2004 on the GDP per capita, in terms of purchasing power parity, were taken from the World Economic Outlook Database [34]. The data for the latest available year on the percentage of rural population with access to improve water source, the GINI, and the income share held by the lowest 10% were obtained from the World Development Indicators [35,36].

The limited availability of socioeconomic data at country level forced the reduction in the number of countries included in the analysis. Starting with 193 countries (i.e. WHO Member States) for consideration, the data on the GDP per capita purchasing power parity (PPP) were available for only 173 countries (see additional file 1). Furthermore, when taking into account income distribution variables, data were available just for 125 countries. The percentage of population that lives with less than 2 dollars per day (PPP) would have been preferable to consider as a measure of absolute poverty, but it was available only for 102 countries. Instead, the variable percentage of rural population with access to clean water was included as a proxy of absolute poverty (allowing 157 observations).

Finally, the data for the year 2004 on the total DALYs and the DALYs for each of the three groups of diseases associated with the burden of disease (i.e. communicable, non-communicable and injuries) were obtained from the WHO Health Statistics and Health Information Systems web site [37]. These data represented an update [38] of the previous global burden of disease analysis [39]. In order to be consistent with the inclusion of a variable, in terms of density per 1000 people, the total DALYs of each category were converted into DALYs per 1000 people using the data on population presented along with the burden of disease data.

The econometric analysis consists of two sets of regression equations with a semi-log functional form. Following Anand and Bärnighausen [21,22], the first set of regressions considers, as an independent variable, the density per 1000 populations for the three categories of health workers aggregated (i.e. physicians, nurses, and midwives). On the other hand, the second set considers the health workers as two different independent variables: the density of physicians and the density of the aggregation of nurses and midwives.

The dependent variables in both sets of equations are the total DALYs per 1000 people and the DALYs per 1000 people for each of the three aforementioned groups of diseases. Considering the limited availability of data for the socioeconomic variables, three different models were estimated for each of the dependent variables; the first one just includes the GDP per capita, the second one includes the GDP and the income distribution variables (GINI and income share held by the lowest 10%), and the third one includes the GDP and the percentage of rural population with access to clear water.

Finally, the variable 'skill mix' was created as the ratio between the number of physicians and the number of nurses and midwives. This variable was included in all the models as a way to capture the effect of the skill mix on the burden of disease. The 'skill mix-squared' term was created as the square of the variable 'skill mix' and was also included in all the models in order to test it for the concavity of the skill mix effect.

The following equations are examples of all the multiple regressions estimated for the dependent variable DALY_ij_, with i the group of disease (0: total; 1: communicable; 2: non-communicable; 3: injuries) and j the country:

### Health workers

$$\begin{array}{cc} \ln({\text{DALY}}_{\text{ij}})= & \begin{array}{l} \beta_{0}+\beta_{1}\cdot Health\_Wor\ker s_{j}+\beta_{2}\cdot GDP_{j}+\\ \beta_{3}\cdot Health\_\exp enditure\_\%\_GDP_{j}+\\ \beta_{4}\cdot Skill\_Mix_{j}+\beta_{5}\cdot Skill\_Mix-Sq_{j} \end{array} \end{array}$$

$$\begin{array}{cc} \text{ln}({\text{DALY}}_{\text{ij}})= & \begin{array}{l} \beta_{0}+\beta_{1}\cdot Health\_Wor\ker s_{j}+\beta_{2}\cdot GDP_{j}+\\ \beta_{3}\cdot Health\_\exp enditure\_\%\_GDP_{j}+\\ \beta_{4}\cdot Skill\_Mix_{j}+\beta_{5}\cdot Skill\_Mix-Sq_{j}+\\ \beta_{6}\cdot GINI_{j}+\beta_{7}\cdot Income\_share\_lowest\_10{\%}_{j} \end{array} \end{array}$$

$$\begin{array}{cc} \text{ln}({\text{DALY}}_{\text{ij}})= & \begin{array}{l} \beta_{0}+\beta_{1}\cdot Health\_Wor\ker s_{j}+\beta_{2}\cdot GDP_{j}\\ \beta_{3}\cdot Health\_\exp enditure\_\%\_GDP_{j}+\\ +\beta_{4}\cdot Skill\_Mix_{j}+\beta_{5}\cdot Skill\_Mix-Sq_{j}+\\ \beta_{6}\cdot \%rural\_population\_access\_clean\_water \end{array} \end{array}$$

### Physicians/nurses and midwives

$$\begin{array}{cc} \text{ln}({\text{DALY}}_{\text{ij}})= & \begin{array}{l} \beta_{0}+\beta_{1}\cdot Physicians_{j}+\beta_{2}\cdot Nurses\_and\_midwives+\\ \beta_{3}\cdot GDP_{j}+\beta_{4}\cdot Health\_\exp enditure\_\%\_GDP_{j}+\\ \beta_{5}\cdot Skill\_Mix_{j}+\beta_{6}\cdot Skill\_Mix-Sq_{j} \end{array} \end{array}$$

$$\begin{array}{cc} \text{ln}({\text{DALY}}_{\text{ij}})= & \begin{array}{l} \beta_{0}+\beta_{1}\cdot Physicians_{j}+\beta_{2}\cdot Nurses\_and\_midwives+\\ \beta_{3}\cdot GDP_{j}+\beta_{4}\cdot Health\_\exp enditure\_\%\_GDP_{j}+\\ \beta_{5}\cdot Skill\_Mix_{j}+\beta_{6}\cdot Skill\_Mix-Sq_{j}+\beta_{7}\cdot GINI+\\ \beta_{8}\cdot Income\_share\_lowest\_10\% \end{array} \end{array}$$

$$\begin{array}{cc} \text{ln}({\text{DALY}}_{\text{ij}})= & \begin{array}{l} \beta_{0}+\beta_{1}\cdot Physicians_{j}+\beta_{2}\cdot Nurses\_and\_midwives+\\ \beta_{3}\cdot GDP_{j}+\beta_{4}\cdot Health\_\exp enditure\_\%\_GDP_{j}+\\ \beta_{5}\cdot Skill\_Mix_{j}+\beta_{6}\cdot Skill\_Mix-Sq_{j}+\\ \beta_{7}\cdot \%rural\_population\_access\_clean\_water \end{array} \end{array}$$

## Results

The additional file 2 shows the statistical description (i.e. number of observation, mean, standard deviation, minimum and maximum) of each one of the dependent and independent variables in general and also separated by WHO region.

All the variables present wide ranges of values, showing the great heterogeneity throughout the countries included in the analysis. For instance, the density of health workers varies between 0.25 (Niger) and 22.4 (Ireland) per 1000 populations, while the number of physicians per 1000 populations goes from 0.02 (Malawi) to 5.9 (Cuba). Furthermore, although on average a country has 0.63 physician per nurse or midwife, when looking to the extremes this number can vary between 0.02 (Swaziland) to 27.54 (The Netherlands) physicians per nurse or midwife.

On the other hand, the differences in terms of burden of disease are also dramatic, from a country with a burden of disease of less than 100 DALYs per 1000 populations (Iceland) to a country that presents a burden of disease almost nine times higher (i.e. 824 DALYs per 1000 populations in Sierra Leone). The same significant differences throughout the countries are observed for the rest of the variables (i.e. health expenditure as percentage GDP, GDP, GINI, income share held by the lowest 10%, and percentage of rural population with access to clean water).

Not surprisingly, when we focus on the regional level, although differences persist within regions, the differences throughout the regions are now much more evident. In general, the most developed regions have better indicators than the regions that consist of the poorest countries (i.e. higher density of health professionals and lower burden of disease). Furthermore, the uneven distribution of health professionals, highly documented in the literature, becomes manifest when we consider that the average density of health workers in Africa is just 1.58 per 1000 while in Europe it is 10.78 per 1000.

**Figure 1:**
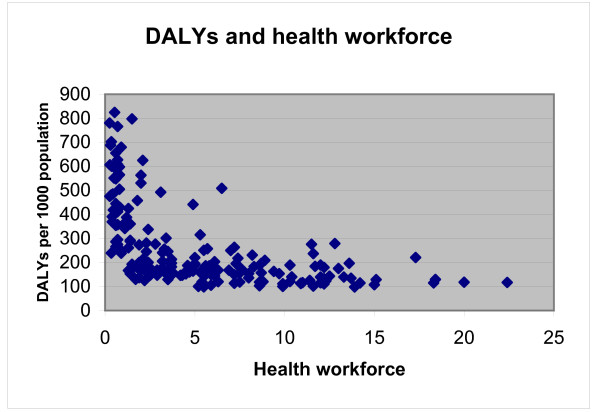
**DALYs and health workers**.

Figure 1 presents the relationship between the health workers and the DALYs for the countries included in the analysis. It is clearly appreciated from the graph that countries with lower relative need (i.e. burden of disease) are actually the countries with a higher number of health professionals. This negative relationship has also been presented in the literature as one of the strong arguments that support the urgent need of scaling up the health workforce [17]. However, this presentation has always been descriptive, therefore the average marginal contribution of an extra health worker in terms of DALY reduction has not been analysed quantitatively. The present study represents a first attempt to measure this relationship.

The Additional file 3 presents the results of the multiple regressions described in the previous section.

In the first set of equations, when we consider the total DALYs (i.e. DALY_0_) as the dependent variable, the results show a negative and a significant effect for the health workers (at 15% in the regression including percentage of access to clean water), the GDP and the Skill Mix. On the other hand, the 'skill mix-squared' had a positive and a significant effect, the percentage of rural population with access to clean water had a negative and a significant effect, while the variables accounting for income distribution (i.e. GINI and income share held by lowest 10%) and health expenditure as percentage of GDP resulted in being not significant. In the second set of equations for the total DALYs, when we consider the models including just GDP as the socioeconomic variable of control and the one including the variables controlling for socioeconomic inequalities, the results show a negative and a significant effect for the variable 'physicians'. However, the 'physicians' variable was found to be not significant in the model controlling for access to clean water. In the three models the variable 'nurses and midwives' was found not to be significant. The sign and the significance of the coefficients for the rest of the variables were the same as in the first set of equations.

In terms of the disaggregation of the dependent variable the results are different depending on the groups of diseases. The coefficients obtained for the group of communicable diseases (i.e. DALY_1 _as the dependent variable) were similar in sign and in significance to the coefficients for the aforementioned total DALYs for the two sets of equations. The only exceptions were the coefficient for 'health workers and physicians', which was negative and significant (at 5%), and the coefficient for the variable GINI which, in the case of this particular group of diseases, was found to be positive and significant.

The findings for the other two groups (i.e. non communicable diseases and injuries) are totally different, not only in terms of significance but surprisingly also in terms of sign. The coefficients for the variables related to human resources are more erratic and less consistent between models than in the case of total DALYs, and the DALYs associated with communicable diseases as dependent variables. In all the cases, the variables accounting for 'health workers and physicians' presented a positive and a significant effect on the DALYs associated with non-communicable diseases. On the other hand, when we considered the DALYs related to injuries, the coefficient for 'health workers' was negative and significant in one of the models of the first set of equations, while the coefficient for 'physicians' resulted in being negative and significant in two of the models, the exception being the model controlling for the percentage of rural population with access to clean water (i.e. with a not significant effect).

For the groups of DALYs related to non-communicable diseases and injuries, the coefficients for the variables 'skill mix' and 'skill mix-squared' was found to be not significant at 5% for any of the models, the same occurred in the case of the variables 'health expenditure as percentage of GDP' and 'income share held by the lowest 10%'. The percentage of rural population with access to clean water resulted in being negative and significant in the two models for the DALYs associated to injuries. The only variable which presented a significant and a consistent behaviour in all the models for these two groups was GDP (i.e. negative in all the cases).

## Discussion

In terms of the strength of the relationship between human resources for health and burden of disease, as the functional form of the equations was semi-log, the coefficients cannot be interpreted directly as elasticities, but as the percentage changes in the dependent variable following a unit change in the independent variable. Considering this, an increase of one unit in the density of health workers per 1000 will decrease, on average, the total burden of disease between 1% and 3%.

Focusing on the group of communicable diseases, which presented the most consistent pattern of results, the health workers seem to play an even more important role. An increase of one unit in the density of health workers per 1000 will decrease, on average, the DALYs associated to this group of diseases between 10% and 15%. Moreover, if the density of physicians per 1000 populations is the one which increases in one unit, the effect is even higher (i.e. between 30 and 45%).

**Figure 2:**
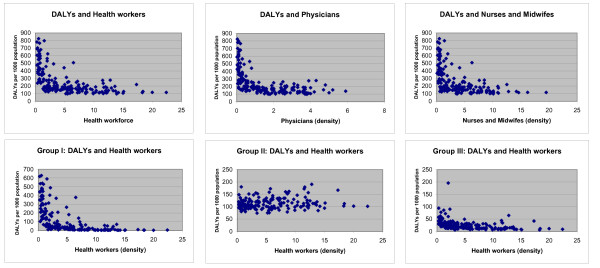
**DALYs and health workers (aggregated and disaggregated)**.

The choice of the functional form may be subject to discussion. Although most of the previous articles state the use of some kind of linear functional form (e.g. log-linear, arcsin-log), and the ones including vaccine coverage or coverage with skilled birth attendants use a logit-log form, the present study opted for the semi-log functional form. The election of a semi-log functional form relies on the idea that the relationship between the independent variables included in the analysis and in the DALYs is not linear. On the other hand, the logit-log forms are appropriate in the case of variables accounting for coverage due to the scale from 0 to 100%, but this is not the case of the DALYs per 1000 variables. The Figure 2 shows a graphic representation of the relationship between the dependent variables for the different models (i.e. DALY_0_, DALY_1_, DALY_2 _and DALY_3_) and the measures of health workers. The graphics show an exponential relationship between them, the main exception being the relationship between the DALYs in the group of non-communicable diseases and health workers.

The aggregate analysis shows that health workers are an important determinant of health outcomes. Even when the functional forms and the health outcomes considered are not necessarily the same, this result is in accordance with previous findings, stating that health workers significantly affect immunisation coverage, infant and under-5 mortality, and the other health outcomes. The main finding presented in this article is that the positive and significant relationship between human resources and health outcomes can be extended to a much broader measure of population health (i.e. DALYs), and that this relationship may follow different patterns according to the different groups of diseases.

The density of nurses and midwives is found to be not significant in most of the models. The same results are presented by Robinson and Wharrad [23] when they measured the relationship between infant and under-5 mortality rates, and the density of nurses. Later Robinson and Wharrad [24] considered attendance at birth and maternal mortality rates. This effect is what the authors called 'invisible nurses'. Anand and Bärninghausen [22], assessing the relationship between nurses and maternal, infant and under-five mortality, found that nurses were significantly associated just with maternal mortality.

The importance of physicians, in contrast to nurses and midwives in the reduction of the burden of disease, is also reaffirmed by the significant and the negative relationship between the independent variable 'skill mix' and the dependent variables 'total DALYs' and 'DALYs related to communicable diseases'. The variable was constructed as the ratio between physicians, nurses, and midwives. Therefore, a negative coefficient implies that the higher the number of physicians, in relation to the number of nurses and midwives, the greater the reduction of DALYs. However, the fact that 'skill mix-squared' presented a positive and a significant association with the total DALYs and DALYs associated with communicable diseases confirms the concavity of the relationship between the DALYs and the ratio physicians/nurses and midwifes, meaning that despite increasing, it increases at a decreasing rate.

As Robinson and Wharrad stated [[24], p.452], the danger related to the 'invisibility' of nurses in the econometric analysis is its contribution 'to the perceived dominance of medicine in the social construction of health services worldwide', underestimating the independent contribution to health care of nursing and midwifery. The article suggests that this is maybe because the quality of the data on these cadres and the ambiguity about the definition of 'registered nurse'. Although the data used in the present study are the best data available, as the processes of collection and homogenisation of data are improving every day, further studies will be able to reassess this finding.

The variable GDP per capita (measured in terms of purchasing power parity) was included in order to capture the effect of socioeconomic determinants of health. It resulted to be the most consistently significant variable, showing, as mentioned in the previous section, that health can be affected by factors beyond the health care system. However, Robinson and Wharrad [[23], p.36] state that 'the use of GDP per capita as a measure of a country's wealth has several limitations', for instance it does not take into account the degree of equity in the distribution of this wealth. The study, trying to overcome this deficiency, included two dependent variables in order to control for income distribution (i.e. 'GINI' and 'income share held by the lowest 10%'). However, these variables did not present a significant relationship with the burden of disease, the only exceptions being the coefficients for the variable GINI when the dependent variables were DALYs associated to communicable and non-communicable diseases, though the effects were opposite (negative and positive respectively). Therefore, the income distribution seems not to have a consistent effect on the burden of disease while the income does have a strong impact. However, this result should be considered cautiously because about fifty countries, mostly developing countries, were excluded from the analysis (see additional file 1). The fact that income distribution, regardless of the exclusion of many countries from the sample, still has a negative impact on the group of communicable is an interesting finding, probably also related to the particularities of this group of diseases (e.g. affecting more poor countries; access to immunization probably related to income distribution).

As an alternative to the models including the income distribution variables, the third type of model included the variable 'percentage of rural population with access to clean water' as a proxy of absolute poverty. When included, the effect of the variable on total DALYs (and DALYs related to the different groups of diseases) always resulted in being negative and significant. This finding shows, as well as with the GDP, the influence of variables beyond the health system on the burden of disease. Furthermore, the inclusion of this proxy of absolute poverty allows us to consider a socioeconomic variable for a larger sample of countries, avoiding the aforementioned possible bias regarding the non availability of socioeconomic inequality data for an important number of countries.

The variable 'health expenditure' as percentage of GDP was included as a way to take into account the health system capacity, but it was consistently found to be not significant. In other words, how much of the total national income is going to health care does not affect population health. As health workers generally account for the most important part of the health budget and variables accounting for health workers and the variable GDP are also included, one possible explanation for the insignificance of the health expenditure as percentage of the GDP could be the multicollinearity. However, the variance inflation factor (VIF) analysis showed that the multicollinearity is not a problem in this case (VIF is lower than 2 for the specific variable and means that VIF is lower than 10 on average considering all the models).

**Figure 3:**
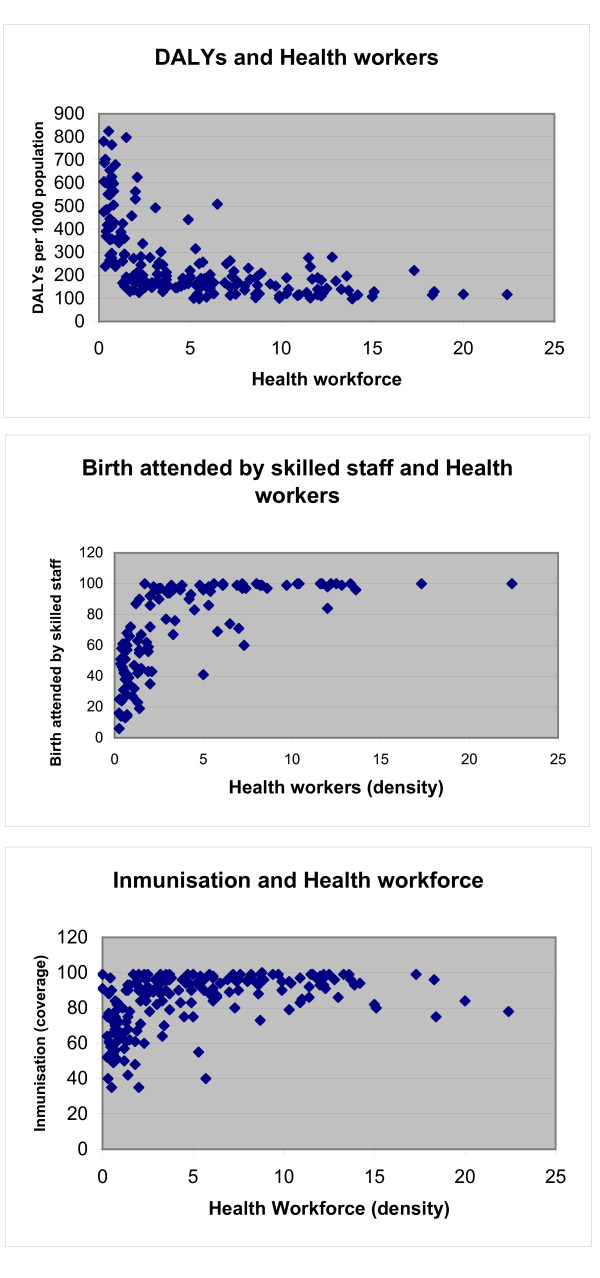
**Health outcomes and health workers**.

The use of DALYs can be criticised as the dependent variable. One of the main disadvantages of DALYs is all the requirements for the estimation. For instance, mortality rates, prevalences and incidences related to specific causes and groups of age, which are not available for all the countries (especially developing countries), should be estimated. On the other hand, there are also assumptions made on the constructions of the DALYs, like the use of a discount rate (and which one to use) or the inclusion of age weights that may change the results obtained. Despite certain criticisms, the methodology used to estimate the DALYs has been improved, and the data used in this study correspond to an update of the previous estimation for the year 2004, with more recent registration data, improvements in methods used to estimate the parameters in countries with unavailable data, and estimations based on epidemiological studies, diseases registers, etc. What is obtained from the briefly aforementioned methodologies is a more comprehensive indicator of health (comparable between regions and countries), as it includes not only mortality but also disability; considering diseases that may not be captured for the health outcomes which were considered in the other studies. Furthermore, the 'variables such as 'coverage of immunization' or 'coverage of skilled birth attendants' as dependent variables have a limit of 100% (see Figure 3) and they could be considered as disadvantage in the case of a cross-sectional analysis. As many countries reached the maximum possible coverage several years ago and the cross-sectional analysis does not take into account lagged relationships, the association between the variables may be weakened. Although the same argument might be applied in the case of DALYs, as burden of disease, in theory, it does not have a limit (below zero): it can always be diminished, even if it is at a decreasing rate.

It was mentioned before that various assumptions are made when we estimate the DALYs. It would be interesting to replicate the analysis proposed by this study considering different sensitivities for DALYs (e.g. discount rate different to 3% or not considering age weights) in order to check them if the results change when the assumptions made on the calculations of DALYs change. However, the data on these different sensitivities are not publicly available at the country level, but just at the regional or groups of income level.

Although the results in terms of significance and direction (i.e. sign) of the relationship between human resources and burden of disease were mainly in accordance with what was expected, especially considering the group of communicable diseases, one interesting finding of the study is the completely different behaviour of the models considering DALYs for non-communicable diseases and injuries as dependent variables. This can probably be explained because of the different nature of the three groups of conditions and also because of the totally different composition of the burden of disease throughout different countries. While non-communicable are the most important causes in developed countries, in developing countries communicable diseases are still the most important. On the other hand, it is intuitively easy to find a link between health care (i.e. health workers) and communicable diseases, but when considering non-communicable diseases or injuries the link appears to be less intuitive and other variables such as life style or existence of specific risk factors in the population arise and take a place into the story.

It is likely, due to the limited availability of data, that some variables have been omitted from the models, especially in the case of the models for the dependent variables for the groups II and III of diseases (i.e. non-communicable and injuries). In these two particular cases, the existence of omitted variables (e.g. life styles and existence of risk factors) may be a possible explanation for the inconsistent results obtained in this study. Further studies are necessary in this area, either to find reasonable explanations for this finding or to improve the methodology in order to find a better model to assess the relationship between health workers and burden of disease related to non-communicable diseases and injuries.

Even though the study presents the limitations mentioned throughout this section (e.g. cross-sectional analysis, availability of data, functional form, and omitted variables) and the results must be interpreted cautiously, it represents a first attempt to relate a broader concept of health to human resources of health. Further researches with improved methodologies are necessary to generate empirical support in order to define most accurate policies in this area.

## Conclusion

The relationship between human resources for health and health outcomes has been analysed mostly considering specific health outcomes such as mortality rate, coverage of vaccination or skilled birth attendance. The effect of health workers on health has been proven to be important for all of the outcomes analysed in the literature, particularly the effect of physicians on health. However, health represents a much broader concept; it includes not only mortality but also morbidity, and not only preventive but also curative or improving quality of life interventions. In this context, the analysis of the relationship between health workers and DALYs represents the first attempt at measuring the link between human resources for health and a more comprehensive health outcome.

This study presents evidence of a statistically negative relationship between the density of health workers (specifically physicians) and the burden of disease when controlling for income and income distribution variables. In terms of magnitudes, an increase of one unit in the density of health workers per 1000 will decrease, on average, the total burden of disease between 1% and 3%. In the case of the density of physicians the impact is even higher: an increase in one unit of this density can decrease, on average, the total DALYs by about 10%. In the case of nursing and midwifery, the findings are that, in accordance with previous articles, the density of these professionals does not affect the DALYs.

The analysis of the three groups of burden of disease showed that the only group that presents the same behaviour as total DALYs, in terms of significance and sign of the coefficients (while the magnitude of the effects are higher), is the group of communicable diseases. For the two other groups, health workers were found not to be significant, even showing the opposite sign (i.e. positive association between health workers and DALYs).

In summary, if countries increase health worker density, they will be able to reduce significantly their burden of disease, especially in the case of communicable diseases. The findings of the study have implications not only for health and health policy, but also for research. They represent supporting evidence of the importance of health workers for health, and therefore they contribute to the development of policies in this area. Furthermore, the study limitations, as well as the unexpected results for some of the variables, encourage future research to improve methodologies and analysis.

## Competing interests

The authors declare that they have no competing interests.

## Supplementary Material

Additional file 1 **Variables and countries with unavailable data**. Click here for file

Additional file 2 **Statistical description (i.e. number of observation, mean, standard deviation, minimum and maximum) of each one of the dependent and independent variables in general and also separated by WHO region**. Click here for file

Additional file 3 **The results of the multiple regressions**. Notes: [_] Standard error; (*) Significant at 5%; (**) Significant at 10%; (***) Significant at 15% Click here for file

## References

[B1] Gupta N Dal Poz M Assessment of human resources for health using cross-national comparison of facility surveys in six countries Human Resources for Health 2009 7 22 10.1186/1478-4491-7-22 19284604 PMC2660277

[B2] The World Health Report 2006 Working together for health 2006 Geneva: World Health Organization

[B3] Hongoro Ch McPake B How to bridge the gap in human resources for health The Lancet 2004 364 1451 1456 10.1016/S0140-6736(04)17229-2 15488222

[B4] Narasimhan V Brown H Pablos-Mendez A Adams O Dussault G Elzinga G Nordstrom A Habte D Jacobs M Solimano G Sewankambo N Wibulpolprasert S Evans T Chen L Responding to the Global Human Resources Crisis The Lancet 2004 363 1469 1472 10.1016/S0140-6736(04)16108-4 15121412

[B5] Chen L Evans T Anand S Boufford JI Brown H Chowdhury M Cueto M Dare L Dussault G Elzinga G Fee E Habte D Hanvoravongchai P Jacobs M Kurowski C Michael S Pablos-Mendez A Sewankambo N Solimano G Stilwell B de Waal A Wibulpolprasert S Human Resources for Health: overcoming the crisis The Lancet 2004 364 1984 1990 10.1016/S0140-6736(04)17482-5 15567015

[B6] Speybroeck N Ebener S Sousa A Paraje G Evans D Prasad A Inequality in access to human resources for health: measurement issues 2006 Geneva: World Health Organization http://www.who.int/hrh/documents/whr06_background_papers/en/index.html (background paper for The World Health Report 2006) 10.1111/j.1538-4632.2012.00842.x PMC3378331 22736806

[B7] Dussault G Dubois CA Human resources for health policies: a critical component in health policies Human Resources for Health 2003 1 1 10.1186/1478-4491-1-1 12904254 PMC166115

[B8] Dräger S Dal Poz M Evans D Health workers wages: an overview from selected countries 2006 Geneva: World Health Organization http://www.who.int/hrh/documents/whr06_background_papers/en/index.html (background paper for The World Health Report 2006

[B9] Vujicic M Zurn P Diallo K Adams O Dal Poz M The role of wages in the migration of health care professionals from developing countries Human Resources for Health 2004 2 3 10.1186/1478-4491-2-3 15115549 PMC419378

[B10] Scheffler R Liu JX Kinfu Y Dal Poz M Forecasting the global shortage of physicians: an economic- and needs-based approach Bulletin of the World Health Organization 2008 86 7 516 523 10.2471/BLT.07.046474 18670663 PMC2647492

[B11] Dovlo D Using mid level cadres as substitutes for internationally mobile health professionals in Africa. A desk review Human Resources for Health 2004 2 7 10.1186/1478-4491-2-7 15207010 PMC455693

[B12] Lehmann U Van Damme W Barten F Sanders D Task shifting: the answer to the human resources crisis in Africa? Human Resources for Health 2009 7 49 10.1186/1478-4491-7-49 19545398 PMC2705665

[B13] Buchan J Dal Poz M Skill mix in the health care workforce: reviewing the evidence Bulletin of the World Health Organization 2002 80 7 575 580 12163922 PMC2567564

[B14] WHO Human Resources for Health Minimum Data Set Geneva 2008

[B15] World Health Organization. World Health Statistics 2009. Health workforce, infrastructure, essential medicines. Table 6 http://www.who.int/whosis/whostat/EN_WHS09_Table6.pdf

[B16] Speybroeck N Kinfu Y Dal Poz M Evans D Reassessing the relationship between human resources for health, intervention coverage and health outcomes 2006 Geneva: World Health Organization http://www.who.int/hrh/documents/whr06_background_papers/en/index.html (background paper for The World Health Report 2006)

[B17] Scaling up health workforce production: a concept paper towards the implementation of World Health Assembly resolution WHZ59.23 2006 Geneva: World Health Organization

[B18] Scheffler R Mahoney C Fulton B Dal Poz M Preker A Estimates of Health Care Professional Shortages in Sub Saharan Africa by 2015 Health Affairs 2009 28 5 849 862 10.1377/hlthaff.28.5.w849 19661111

[B19] Diallo K Zurn P Gupta N Dal Poz M Monitoring and evaluation of human resources for health: an international perspective Human Resources for Health 2003 1 3 10.1186/1478-4491-1-3 12904252 PMC179874

[B20] Smith PC Mossialos E Papanicolas Performance measurement for health system improvement: experiences, challenges and prospects 2008 Denmark: World Health Organization Europe

[B21] Anand S Bärnighausen T Health workers and vaccination coverage in developing countries: an econometric analysis The Lancet 2007 369 1277 1285 10.1016/S0140-6736(07)60599-6 17434403

[B22] Anand S Bärnighausen T Human resources and health outcomes: cross-country econometric study The Lancet 2004 364 1603 1609 10.1016/S0140-6736(04)17313-3 15519630

[B23] Robinson J Wharrad H Invisible nursing: exploring health outcomes at a global level--relationships between infant and under-5 mortality rates and the distribution of health professionals, GNP per capita, and female literacy Journal of Advanced Nursing 2000 32 1 28 40 10.1046/j.1365-2648.2000.01458.x 10886432

[B24] Robinson J Wharrad H The relationship between attendance at birth and maternal mortality rates: an exploration of United Nations' data sets including the ratios of physicians and nurses to population, GNP per capita and female literacy Journal of Advanced Nursing 2001 34 4 445 455 10.1046/j.1365-2648.2001.01773.x 11380711

[B25] Kim K Moody PM More resources better health? A cross-national perspective Social Science and Medicine 1992 34 8 837 842 10.1016/0277-9536(92)90253-M 1604375

[B26] Hertz E Hebert JR Landon J Social and environmental factors and life expectancy, infant mortality, and maternal mortality rates: results of a cross-national comparison Social Science and Medicine 1994 39 105 114 10.1016/0277-9536(94)90170-8 8066481

[B27] Cochrane AL St Leger AS Moore F Health service 'input' and mortality 'output' in developed countries Journal of Epidemiology and Community Health 1997 51 344 348 10.1136/jech.51.4.344 9379140 PMC1060897

[B28] Nolte E Bain C McKee M Smith PC, Mossialos E, Leatherman S, Papanicolas I Population Health Performance Measurement for Health System Improvement: Experiences, Challenges and Prospects 2009 1 Cambridge: Cambridge University Press 27 62

[B29] Wilkinson R, Marmot M Social determinants of health: the solid facts 2003 2 Denmark: World Health Organization Europe

[B30] Marmot M Social determinants of health inequalities The Lancet 2005 365 1099 1104 10.1016/S0140-6736(05)71146-6 15781105

[B31] Dal Poz M Kinfu Y Drager S Kunjumen T Counting health workers: definitions, data, methods and global results 2006 Geneva: World Health Organization http://www.who.int/hrh/documents/whr06_background_papers/en/index.html (background paper for The World Health Report 2006)

[B32] Global Health Atlas http://apps.who.int/globalatlas/DataQuery/default.asp

[B33] Xu K Evans D Kawabata K Zeramdini R Klavus J Murray C Household catastrophic health expenditure: a multicountry analysis The Lancet 2003 362 111 117 10.1016/S0140-6736(03)13861-5 12867110

[B34] World Economic Outlook Database 2004 http://www.imf.org/external/pubs/ft/weo/2004/02/data/dbginim.cfm

[B35] World Development Indicators. Improved water source, rural (% of rural population with access) http://data.worldbank.org/indicator/SH.H2O.SAFE.RU.ZS

[B36] 2007 World Development Indicators. Distribution of Income or Consumption. Table 2.7 http://siteresources.worldbank.org/DATASTATISTICS/Resources/table2_7.pdf

[B37] Health Statistics and Health Information Systems. Death and DALY estimates for 2004 by cause for WHO Member States http://www.who.int/healthinfo/global_burden_disease/estimates_country/en/index.html

[B38] The Global Burden of Disease 2008 Geneva: World Health Organization 2004 Update

[B39] Global burden of disease and risk factors Lopez AD, Mathers CD, Ezzati M, Jamison DT, Murray CJL 2006 New York, Washington, DC: Oxford University Press; World Bank

