# Supplementary material for: Human resources for health and burden of disease: an econometric approach
Source: Hum Resour Health. 2011 Jan 26;9:4. doi: 10.1186/1478-4491-9-4 (PMC3039562; doi:10.1186/1478-4491-9-4)
Supplement: Additional file 1 — Variables and countries with unavailable data. [file 1478-4491-9-4-S1.DOC]

**Variables and countries with unavailable data**

**DALYs:** Montenegro, Serbia.

**Health workers**: Monaco, San Marino, Serbia.

**GDP:** Afghanistan, Andorra, Cook Islands, Cuba, Democratic People’s Republic of Korea, Iraq, Liberia, Libyan Arab Jamahiriya, Marshall Islands, Micronesia (Federated States of), Monaco, Montenegro, Nauru, Niue, Palau, San Marino, Serbia, Somalia, Timor-Leste, Tuvalu.

**GINI and Con_ 10:** Afghanistan, Andorra, Angola, Antigua and Barbuda, Bahamas, Bahrain, Barbados, Belize, Bhutan, Brunei Darussalam, Cape Verde, Chad, Comoros, Congo, Cook Islands, Cuba, Cyprus, Democratic People’s Republic of Korea, Democratic Republic of the Congo, Djibouti, Dominica, Equatorial Guinea, Eritrea, Fiji, Gabon, Grenada, Guyana, Iceland, Iraq, Kiribati, Kuwait, Lebanon, Liberia, Libyan Arab Jamahiriya, Luxembourg, Maldives, Malta, Marshall Islands, Mauritius, Micronesia (Federated States of), Monaco, Montenegro, Myanmar, Nauru, Niue, Oman, Palau, Qatar, Saint Kitts and Nevis, Saint Lucia, Saint Vincent and the Grenadines, Samoa, San Marino, Sao Tome and Principe, Saudi Arabia, Serbia, Seychelles, Solomon Islands, Somalia, Sudan, Suriname, Syrian Arab Republic, Timor-Leste, Togo, Tonga, Tuvalu, United Arab Emirates, Vanuatu.

**Percentage of rural population with access to improved water source:** Bahrain, Belgium, Brunei Darussalam, Cook Islands, Ireland, Italy, Kuwait, Lithuania, Monaco, Nauru, New Zealand, Niue, Poland, Saint Vincent and the Grenadines, San Marino, Saudi Arabia, Seychelles, Singapore, Slovenia, Turkmenistan, Tuvalu, Venezuela (Bolivarian Republic of).
