# Supplementary material for: Human resources for health and burden of disease: an econometric approach
Source: Hum Resour Health. 2011 Jan 26;9:4. doi: 10.1186/1478-4491-9-4 (PMC3039562; doi:10.1186/1478-4491-9-4)
Supplement: Additional file 2 — Statistical description (i.e. number of observation, mean, standard deviation, minimum and maximum) of each one of the dependent and independent variables in general and also separated by WHO region. [file 1478-4491-9-4-S2.PDF]

Variable	Obs	Mean	Std. Dev.	Min	Max
**Health Workers per 1000**	191	5.259476	4.642743	0.25	22.4
**Physicians per 1000**	191	1.490366	1.384903	0.02	5.9
**Nurses and Midwives per 1000**	191	3.76911	3.561939	0.1	19.5
**DALY per 1000**	192	257.4333	167.1931	98.03297	824.4375
**DALY group 1 per 1000**	191	118.4955	154.2416	4.114801	628.6608
**DALY group 2 per 1000**	191	112.7234	21.43758	73.94587	190.5451
**DALY group 3 per 1000**	191	26.68256	20.39845	5.516731	195.7824
**GDP per capita**	173	9398.356	10146.19	591.945	61815.93
**Health Expenditure as % of GDP**	190	6.252632	2.372543	2	15
**Skill Mix**	191	0.6373448	2.044625	0.0250439	27.5462
**GINI**	125	40.8024	10.03929	24.7	74.3
**Income share held by lowest 10%**	125	2.40192	0.9940352	0.3	4.8
**% Rural population with access to clean water**	171	76.94152	22.12922	10	100

Health Workers

WHO Region	Obs	Mean	Std. Dev.	Min	Max
**African Region**	46	1.589348	1.885332	0.25	9.4
**European Region**	51	10.78235	3.875962	4.5	22.4
**Eastern Mediterranean Region**	21	3.62381	2.603825	0.3	8.8
**Region of the Americas**	35	4.222857	2.945893	0.4	13.3
**South-East Asia Region**	11	2.240909	2.016286	0.35	7.4
**Western Pacific Region**	27	4.925926	3.620411	0.6	13

Physicians per 1000

WHO Region	Obs	Mean	Std. Dev.	Min	Max
**African Region**	46	0.235	0.3097078	0.02	1.5
**European Region**	51	3.152941	0.8697938	1.2	5
**Eastern Mediterranean Region**	21	1.247619	0.8863516	0.1	2.7
**Region of the Americas**	35	1.505714	1.254156	0.2	5.9
**South-East Asia Region**	11	0.6318182	0.9220284	0.05	3.3
**Western Pacific Region**	27	1.007407	0.7710761	0.1	2.6

Nurses and Midwives per 1000

WHO Region	Obs	Mean	Std. Dev.	Min	Max
**African Region**	46	1.354348	1.652971	0.2	7.9
**European Region**	51	7.629412	3.520926	2.9	19.5
**Eastern Mediterranean Region**	21	2.37619	1.831094	0.2	6.1
**Region of the Americas**	35	2.717143	2.362512	0.1	10.1
**South-East Asia Region**	11	1.609091	1.222665	0.3	4.1
**Western Pacific Region**	27	3.918518	3.01535	0.5	11

DALY per 1000

WHO Region	Obs	Mean	Std. Dev.	Min	Max
**African Region**	46	474.0548	166.8764	161.1392	824.4375
**European Region**	52	157.4495	47.56066	98.03297	278.8466
**Eastern Mediterranean Region**	21	253.5408	194.1233	98.28874	765.776
**Region of the Americas**	35	182.3696	46.83841	115.3115	369.107
**South-East Asia Region**	11	249.8456	33.34223	199.5171	295.1414
**Western Pacific Region**	27	184.3597	65.19774	101.6983	364.6424

DALY group 1 per 1000

WHO Region	Obs	Mean	Std. Dev.	Min	Max
African Region	46	332.5609	149.609	25.16511	628.6608
European Region	51	18.13081	26.64736	4.114801	142.45
Eastern Mediterranean Region	21	109.143	145.1017	10.90659	540.1978
Region of the Americas	35	44.0915	37.16645	5.184091	210.3672
South-East Asia Region	11	98.01442	42.85665	30.23285	163.1983
Western Pacific Region	27	55.43723	48.76599	4.77295	211.5915

DALY group 2 per 1000

WHO Region	Obs	Mean	Std. Dev.	Min	Max
African Region	46	104.1366	14.80299	73.94587	136.1438
European Region	51	122.4525	25.47496	84.34432	190.5451
Eastern Mediterranean Region	21	106.572	26.90042	74.92654	180.1374
Region of the Americas	35	113.452	13.52116	92.90752	147.5196
South-East Asia Region	11	114.6332	11.01149	93.40784	132.222
Western Pacific Region	27	112.0375	23.22346	85.77001	172.8048

DALY group 3 per 1000

WHO Region	Obs	Mean	Std. Dev.	Min	Max
African Region	46	37.3573	14.01935	14.01269	77.6552
European Region	51	16.65896	11.81385	5.516731	64.71962
Eastern Mediterranean Region	21	37.82579	42.7891	11.6285	195.7824
Region of the Americas	35	24.82606	10.86761	9.035224	60.27202
South-East Asia Region	11	37.19807	19.96858	17.23864	90.13053
Western Pacific Region	27	16.88497	8.115379	5.97575	40.01312

GDP per capita

WHO Region	Obs	Mean	Std. Dev.	Min	Max
African Region	45	2981.373	3565.505	591.945	15216.02
European Region	48	17111.69	12652.77	1028.835	61815.93
Eastern Mediterranean Region	17	8673.925	7429.326	692.651	26038.42
Region of the Americas	34	8785.024	7687.82	1618.21	38031.43
South-East Asia Region	9	3575.51	2232.251	1327.536	7444.165
Western Pacific Region	20	9603.275	9495.195	1695.926	28704.71

Health Expenditure as % of GDP

WHO Region	Obs	Mean	Std. Dev.	Min	Max
African Region	46	5.369565	2.132903	2	11
European Region	51	7.27451	2.050155	3	11
Eastern Mediterranean Region	20	5	2.406133	2	12
Region of the Americas	35	7.028571	2.148676	4	15
South-East Asia Region	11	4.818182	2.136267	2	10
Western Pacific Region	27	6.333333	2.572039	3	12

Skill Mix

WHO Region	Obs	Mean	Std. Dev.	Min	Max
African Region	46	0.1789856	0.1629023	0.0250439	0.9187423
European Region	51	1.011256	3.796483	0.1702723	27.5462
Eastern Mediterranean Region	21	0.6495618	0.5601047	0.2086137	2.666737
Region of the Americas	35	1.014666	1.048345	0.0515021	4.3
South-East Asia Region	11	0.3872057	0.3157343	0.0440111	1.086393
Western Pacific Region	27	0.3152634	0.3117653	0.0769231	1.47917

GINI

WHO Region	Obs	Mean	Std. Dev.	Min	Max
African Region	32	46.8375	9.755586	30	74.3
European Region	44	32.69773	4.891164	24.7	43.6
Eastern Mediterranean Region	7	37.07143	4.358407	30.6	43
Region of the Americas	23	50.6087	7.145432	32.6	60.1
South-East Asia Region	6	38.98333	5.214755	33.4	47.2
Western Pacific Region	13	38.87692	7.705318	24.9	50.9

Income share held by the lowest 10%

WHO Region	Obs	Mean	Std. Dev.	Min	Max
African Region	32	1.996875	0.8380024	0.5	3.9
European Region	44	3.031818	0.6189912	2	4.3
Eastern Mediterranean Region	7	2.9	0.7257181	2	4
Region of the Americas	23	1.258261	0.624992	0.3	2.6
South-East Asia Region	6	3.2	0.4939636	2.6	3.7
Western Pacific Region	13	2.653846	1.005498	1.6	4.8

% Rural population with access to clean water

WHO Region	Obs	Mean	Std. Dev.	Min	Max
African Region	45	56.8	19.09855	26	100
European Region	44	95.06818	9.810762	58	100
Eastern Mediterranean Region	18	71.77778	26.34289	10	100
Region of the Americas	33	82.54545	14.09586	51	100
South-East Asia Region	11	80.90909	12.07853	56	100
Western Pacific Region	20	75.6	21.77493	32	100
