# Supplementary material for: Human resources for health and burden of disease: an econometric approach
Source: Hum Resour Health. 2011 Jan 26;9:4. doi: 10.1186/1478-4491-9-4 (PMC3039562; doi:10.1186/1478-4491-9-4)
Supplement: Additional file 3 — The results of the multiple regressions. Notes: [_] Standard error; (*) Significant at 5%; (**) Significant at 10%; (***) Significant at 15% [file 1478-4491-9-4-S3.PDF]

Semi-log	ln(DALY0)						ln(DALY1)					
	Health Workers + Socioeconomics Inequalities		Health Workers + Access to water (rural)		Health Workers		Health Workers + Socioeconomics Inequalities		Health Workers + Access to water (rural)		Health Workers	
Health Workers per 1000	-0.0260656	(*)	-0.0139531	(***)	-0.0364705	(*)	-0.1032103	(*)	-0.1090865	(*)	-0.151278	(*)
	[0.0106921]		[0.0094803]		[0.0093559]		[0.022976]		[0.0194388]		[0.0197074]	
GDP per capita	-0.0000253	(*)	-0.0000157	(*)	-0.0000233	(*)	-0.0000633	(*)	-0.0000427	(*)	-0.0000574	(*)
	[0.00000585]		[0.00000402]		[0.00000441]		[0.0000126]		[0.00000824]		[0.00000928]	
Health expenditure as % of GDP	0.0087343		0.0142153		0.0140444		-0.0174423		0.0010452		-0.0091708	
	[0.017862]		[0.0126168]		[0.0139128]		[0.0383833]		[0.02587]		[0.0293062]	
Skill Mix	-0.3287123	(*)	-0.1921235	(*)	-0.2866288	(*)	-0.7096987	(*)	-0.4519381	(*)	-0.6492559	(*)
	[0.054155]		[0.0480601]		[0.0525768]		[0.1163725]		[0.0985442]		[0.110749]	
Skill Mix-squared	0.012042	(*)	0.0069527	(*)	0.010579	(*)	0.026775	(*)	0.0170863	(*)	0.0245895	(*)
	[0.0019971]		[0.0017934]		[0.0019599]		[0.0042915]		[0.0036772]		[0.0041284]	
GINI	0.0107514						0.0591709	(*)				
	[0.0087496]						[0.0188019]					
Income share held by lowest 10%	0.0369914						0.2826521	(***)				
	[0.0815807]						[0.175307]					
% of rural population with access to clean water			-0.0123719	(*)					-0.0254921	(*)		
			[0.0016674]						[0.0034189]			
Constant	5.3825	(*)	6.591918	(*)	5.841853	(*)	2.359916	(**)	7.032897	(*)	5.524397	(*)
	[0.571603]		[0.1203027]		[0.0841927]		[1.228306]		[0.246673]		[0.1773454]	
N	125		157		173		125		157		173	
Adj R-Squared	0.6025		0.6422		0.5214		0.7747		0.7954		0.7164	

Semi-log	ln(DALY2)						ln(DALY3)					
	Health Workers + Socioeconomics Inequalities		Health Workers + Access to water (rural)		Health Workers		Health Workers + Socioeconomics Inequalities		Health Workers + Access to water (rural)		Health Workers	
Health Workers per 1000	0.0259226	(*)	0.0256643	(*)	0.0253712	(*)	-0.0126741		-0.0205158	(***)	-0.027445	(*)
	[0.0042805]		[0.0045516]		[0.00388]		[0.0128239]		[0.0134359]		[0.0115479]	
GDP per capita	-0.0000138	(*)	-0.0000101	(*)	-0.000011	(*)	-0.0000311	(*)	-0.0000192	(*)	-0.0000257	(*)
	[0.00000234]		[0.00000193]		[0.00000183]		[0.00000702]		[0.00000569]		[0.00000544]	
Health expenditure as % of GDP	-0.000409		-0.0023856		0.0019384		-0.0101683		-0.0131799		-0.0130127	
	[0.007151]		[0.0060575]		[0.0057699]		[0.0214233]		[0.017881]		[0.0171726]	
Skill Mix	0.0358894	(**)	0.0178803		0.0188176		-0.1163725	(**)	-0.0167607		-0.0544422	
	[0.0216807]		[0.0230743]		[0.0218046]		[0.0649523]		[0.0681124]		[0.0648955]	
Skill Mix-squared	-0.0015616	(**)	-0.0009222		-0.0009222		0.0036346	(***)	-1.15E-06		0.0014473	
	[0.0007995]		[0.000861]		[0.0008128]		[0.0023953]		[0.0025416]		[0.0024191]	
GINI	-0.0080575	(*)					0.0114907					
	[0.0035029]						[0.0104941]					
Income share held by lowest 10%	-0.0424616						0.0401256					
	[0.0326604]						[0.0978461]					
% of rural population with access to clean water			0.0003779						-0.0064433	(*)		
			[0.0008005]						[0.0023631]			
Constant	5.109568	(*)	4.645932	(*)	4.650078	(*)	3.029709	(*)	3.967724	(*)	3.573949	(*)
	[0.2288385]		[0.0577589]		[0.0349163]		[0.685568]		[0.170497]		[0.103919]	
N	125		157		173		125		157		173	
Adj R-Squared	0.335		0.1915		0.1973		0.5151		0.4114		0.4074	

Semi-log	ln(DALY0)						ln(DALY1)					
	Physicians + Nurses + Midwives + Socioeconomics inequalities		Physicians + Nurses + Midwives + Access to water (rural)		Physicians + Nurses + Midwives		Physicians + Nurses + Midwives + Socioeconomics inequalities		Physicians + Nurses + Midwives + Access to water (rural)		Physicians + Nurses + Midwives	
**Physicians per 1000**	-0.1177717	(*)	-0.0370913		-0.105417	(*)	-0.4508652	(*)	-0.320011	(*)	-0.4637568	(*)
	[0.0434974]		[0.0396756]		[0.0395576]		[0.0893623]		[0.0794825]		[0.0803427]	
**Nurses and Midwives per 1000**	0.0065751		-0.004671		-0.0100325		0.0205296		-0.0244726		-0.0314557	
	[0.0183426]		[0.01814]		[0.017429]		[0.0376835]		[0.0363401]		[0.0353989]	
	-0.0000292	(*)	-0.0000163	(*)	-0.0000247	(*)	-0.000078	(*)	-0.000048	(*)	-0.0000639	(*)
GDP per capita	[0.00000603]		[0.00000414]		[0.00000445]		[0.0000124]		[0.00000829]		[0.00000904]	
Health expenditure as % of GDP	0.0129928		0.0140583		0.0144826		-0.0012983		-0.000386		-0.0071848	
	[0.0176934]		[0.0126465]		[0.0138236]		[0.0363499]		[0.0253348]		[0.0280762]	
	-0.2318162	(*)	-0.1644448	(*)	-0.1995231	(*)	-0.3423685	(*)	-0.1996238	(***)	-0.2544764	(**)
Skill Mix	[0.0695046]		[0.0666563]		[0.0713293]		[0.1427924]		[0.1335333]		[0.1448721]	
	0.008389	(*)	0.0059089	(*)	0.0073253	(*)	0.0129265	(*)	0.0075704	(***)	0.0098433	(**)
Skill Mix-squared	[0.0025869]		[0.0025]		[0.0026615]		[0.0053145]		[0.0050083]		[0.0054055]	
	0.0045422						0.035632	(**)				
GINI	[0.0090753]						[0.0186446]					
Income share held by lowest 10%	-0.0066439						0.1172323					
	[0.0827859]						[0.1700779]					
% of rural population with access to clean water			-0.0122218	(*)					-0.0241242	(*)		
			[0.0016895]						[0.0033847]			
	5.718861	(*)	6.573801	(*)	5.81458	(*)	3.635049	(*)	6.867746	(*)	5.400792	(*)
Constant	[0.583627]		[0.1242755]		[0.0850114]		[1.19902]		[0.2489623]		[0.172661]	
N	125		157		173		125		157		173	
Adj R-Squared	0.6148		0.6407		0.5277		0.8005		0.8039		0.7398	

Semi-log	ln(DALY2)						ln(DALY3)					
	**Physicians + Nurses + Midwives + Socioeconomics inequalities**		**Physicians + Nurses + Midwives + Access to water (rural)**		**Physicians + Nurses + Midwives**		**Physicians + Nurses + Midwives + Socioeconomics inequalities**		**Physicians + Nurses + Midwives + Access to water (rural)**		**Physicians + Nurses + Midwives**	
**Physicians per 1000**	0.0683498	(*)	0.0436956	(*)	0.052483	(*)	-0.1107589	(*)	-0.0753983		-0.0969645	(*)
	[0.0172947]		[0.019011]		[0.0164213]		[0.0523867]		[0.0561068]		[0.0489829]	
**Nurses and Midwives per 1000**	0.0108216	(***)	0.0184309	(*)	0.014975	(*)	0.0222369		0.0015007		-0.0007872	
	[0.0072931]		[0.008692]		[0.0072352]		[0.0220912]		[0.0256525]		[0.0215818]	
GDP per capita	-0.000012	(*)	-9.64E-06	(*)	-0.0000104	(*)	-0.0000353	(*)	-0.0000206	(*)	-0.0000271	(*)
	[0.0000024]		[0.00000198]		[0.00000185]		[0.00000726]		[0.00000585]		[0.00000551]	
Health expenditure as % of GDP	-0.0023792		-0.0022632		0.0017661		-0.0056136		-0.0135523		-0.0125708	
	[0.007035]		[0.0060597]		[0.0057385]		[0.0213094]		[0.0178839]		[0.0171173]	
Skill Mix	-0.0089389		-0.0036893		-0.0154348		-0.0127367		0.0488914		0.0333874	
	[0.0276352]		[0.0319392]		[0.0296105]		[0.083709]		[0.0942613]		[0.0883249]	
Skill Mix-squared	0.0001285		-0.0001087		0.0003572		-0.0002725		-0.0024772		-0.0018334	
	[0.0010285]		[0.0011979]		[0.0011048]		[0.0031155]		[0.0035354]		[0.0032956]	
GINI	-0.0051848	(***)					0.0048496					
	[0.0036084]						[0.01093]					
Income share held by lowest 10%	-0.0222741						-0.0065447					
	[0.0329159]						[0.0997045]					
% of rural population with access to clean water			0.0002609						-0.0060874	(*)		
			[0.0008096]						[0.0023893]			
Constant	4.953953	(*)	4.660051	(*)	4.660803	(*)	3.389466	(*)	3.924752	(*)	3.546449	(*)
	[0.2320517]		[0.0595481]		[0.0352903]		[0.7029002]		[0.1757429]		[0.1052671]	
N	125		157		173		125		157		173	
Adj R-Squared	0.3643		0.1913		0.2063		0.5261		0.4115		0.4114	
